# Atraumatic Anterior Dislocation of the Hip Joint

**DOI:** 10.1155/2015/120796

**Published:** 2015-12-24

**Authors:** Tadahiko Ohtsuru, Yasuyuki Morita, Yasuaki Murata, Junya Itou, Yuji Morita, Yutaro Munakata, Yoshiharu Kato

**Affiliations:** ^1^Department of Orthopedic Surgery, Tokyo Women's Medical University, 8-1 Kawada-Cho, Shinjuku Ward, Tokyo 162-8666, Japan; ^2^Department of Radiology, Tokyo Women's Medical University, Tokyo 162-8666, Japan

## Abstract

Dislocation of the hip joint in adults is usually caused by high-energy trauma such as road traffic accidents or falls from heights. Posterior dislocation is observed in most cases. However, atraumatic anterior dislocation of the hip joint is extremely rare. We present a case of atraumatic anterior dislocation of the hip joint that was induced by an activity of daily living. The possible causes of this dislocation were anterior capsule insufficiency due to developmental dysplasia of the hip, posterior pelvic tilt following thoracolumbar kyphosis due to vertebral fracture, and acetabular anterior coverage changes by postural factor. Acetabular anterior coverage changes in the sagittal plane were measured using a tomosynthesis imaging system. This system was useful for elucidation of the dislocation mechanism in the present case.

## 1. Introduction

Dislocation of the hip joint in adults is usually caused by high-energy trauma such as road traffic accidents or severe falls from heights. Posterior dislocation is observed in most cases, and anterior dislocation is rare [1–3]. However, atraumatic anterior dislocation of the hip joint is extremely rare. We report a case of atraumatic anterior dislocation of the hip joint, along with a review of the literature. The patient and her family were informed about this report and agreed to its publication.

## 2. Case Presentation

A 75-year-old woman complained of pain in her right hip. She had no history of trauma or other diseases. When she stood on her tiptoes and extended her hip joints to pick nuts from a high place, she developed right hip pain and fell. She was transported to the hospital by ambulance.

**Figure 1:**
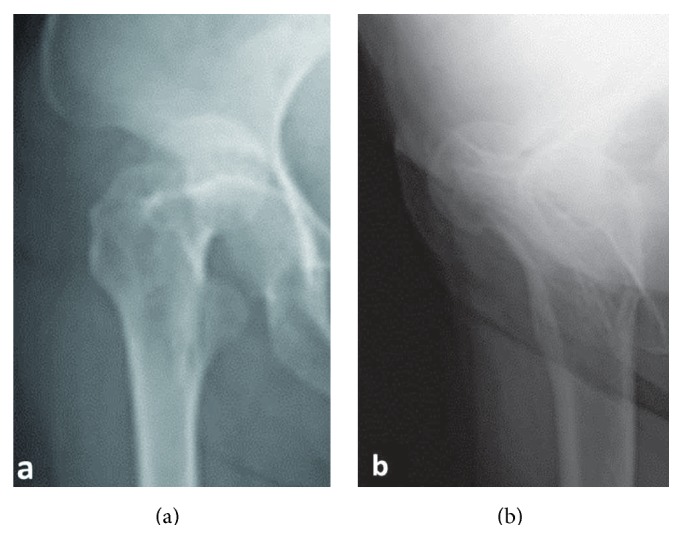
Plain radiographs of the hip joints at the initial visit. (a) Anteroposterior image. (b) Lateral image. These images show anterior dislocation of the right hip joint.

**Figure 2:**
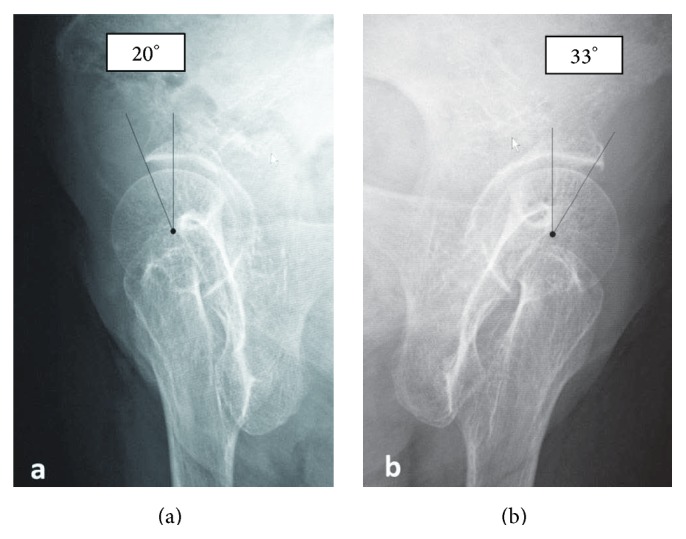
False-profile view of the bilateral hip joint. (a) Right hip joint. (b) Left hip joint. The right and left VCA angles are 20° and 33°, respectively. These findings reveal developmental dysplasia of the right hip joint.

**Figure 3:**
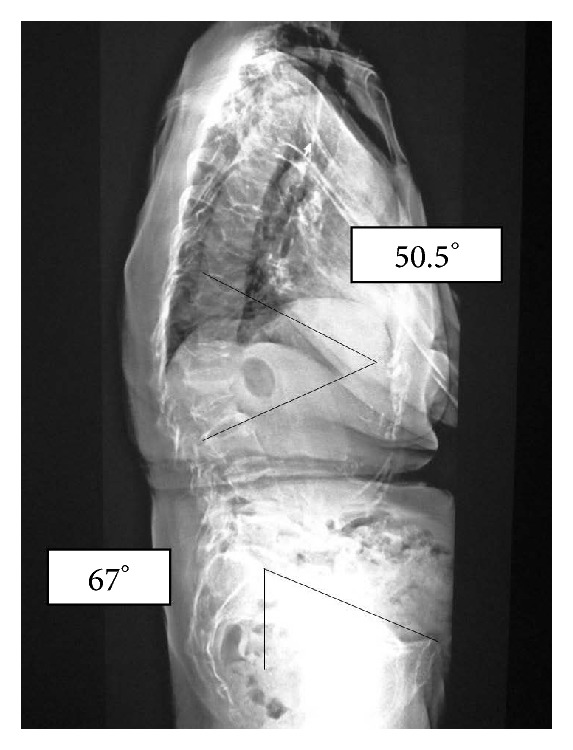
Slot radiography of the spine in the sagittal plane in a standing position. The T10-L2 thoracolumbar angle is 50.5°. Sagittal pelvic tilt angle is 67°. This image reveals severe posterior pelvic tilt following thoracolumbar kyphosis due to vertebral fractures.

**Figure 4:**
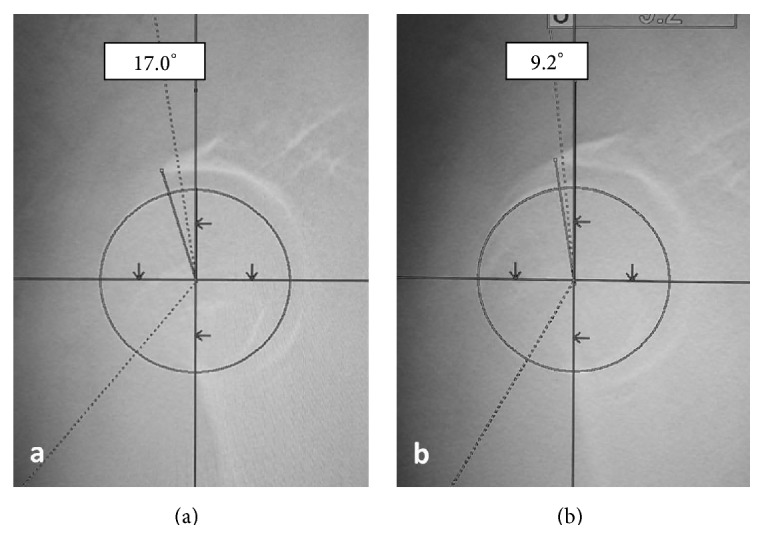
Tomosynthesis of the right hip joint in the sagittal plane in a standing position. (a) The VCA angle is 17.0° in a natural standing position. (b) The VCA angle is 9.2° while standing on tiptoes with hip extension.

**Figure 5:**
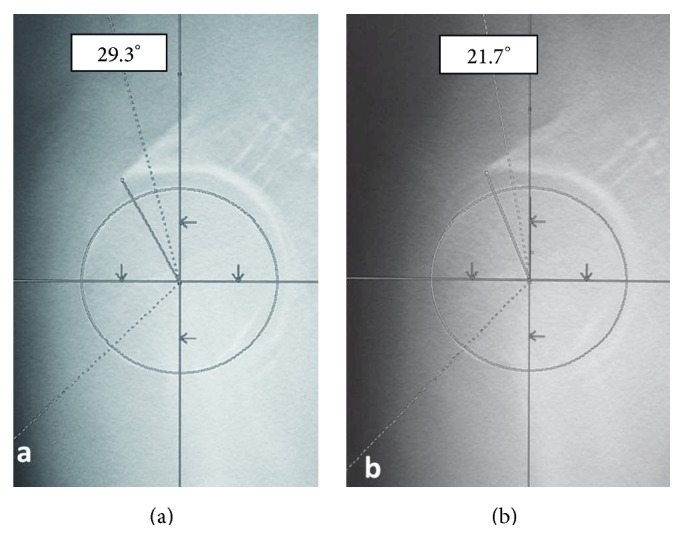
Tomosynthesis of the left hip joint in the sagittal plane in a standing position. (a) The VCA angle is 29.3° in a natural standing position. (b) The VCA angle is 21.7° while standing on tiptoes with hip extension.

On initial examination, she was unable to move her right hip joint; it was fixed at 0° flexion, 30° external rotation, and 10° abduction. A plain radiograph revealed anterior dislocation of the right hip joint (Figure 1). We performed a closed reduction using intravenous anesthetic 4 hours after trauma. The center-edge angles were 34° and 35° in the right and left hip joints, respectively, in a plain radiograph after closed reduction. These angles were normal. However, the vertical center anterior margin (VCA) angle was 20° on the right and 33° on the left in the false-profile view (Figure 2); normal values are greater than 25° [4]. These findings revealed developmental dysplasia of the right hip. Slot radiography of the spine in the sagittal plane in a standing position revealed vertebral fractures of the thoracolumbar spine (Figure 3). The T10-L2 thoracolumbar angle was 50.5°, compared to the normal mean angle of 8.6° [5]. This finding suggested thoracolumbar kyphosis. The sagittal pelvic tilt angle was 67° (the angle between a line connecting the sacral promontory to the pubic symphysis and the vertical line; the normal tilt angle is 19.47 ± 6.26° in men and 24.4 ± 5.93° in women [6]). This finding indicated severe posterior pelvic tilt. In the fluoroscopic examination, her lower extremity was extended manually and showed no mobility in the femoral head. Because she could resist the manipulation, we judged that there was no severe muscle atrophy around her hip joint. Computed tomography (CT) of the hip joints (axial plane) revealed no fracture or free body of bone. Furthermore, the bilateral femoral neck anteversion angles were −5° in both the right and left sides. These findings revealed a retroversion deformity of the proximal femur. Acetabular anterior coverage in the sagittal plane in a natural standing position and standing on tiptoes with hip extension was measured with a tomosynthesis imaging system (SONIALVISION safire with a flat panel detector of 17 × 17, Shimadzu Corporation, Kyoto, Japan). First, bilateral femoral heads were positioned perpendicular to horizontal line of the image under fluoroscopy. A tomosynthesis image was then taken with the beam centered on the femoral head. Angle measurement was performed with a three-dimensional work station (Virtual Place Lexus 64: AZE, Tokyo, Japan). Similar to analysis of the false-profile view, we measured the VCA angles at the slice where the anterior edge of the acetabulum was seen most clearly; the VCA angle of right hip joint was 17.0° in a natural standing position and 9.2° while standing on tiptoes with hip extension (Figure 4), and it was 29.3° and 21.7°, respectively, in the left hip joint (Figure 5).

General joint laxity was evaluated according to scores described by Carter and Wilkinson [7], with negative results. There were no abnormal values on laboratory examination. Bone mineral density (BMD) of the lumber spine (L1-4, 0.61 g/cm^2^, *T*-score: −2.68 SD) measured with dual energy X-ray absorptiometry indicated the presence of associated osteoporosis.

After reduction, the patient started partial weight-bearing walking exercises wearing a hip brace. Full weight-bearing walking exercises were started one month after reduction. A parathyroid hormone formulation was administered for treating osteoporosis.

On her final follow-up one year after the reduction, the patient was free from pain and was able to walk freely. She had full range of hip motion. Magnetic resonance imaging of the hip joints (T1-weighted image) revealed no osteonecrosis of the femoral head. The Harris hip score improved to 92. She has had no recurrence of dislocation and was extremely satisfied.

## 3. Discussion

Dislocation of the hip joint in adults is usually caused by high-energy trauma such as road traffic accidents or fall from heights. Atraumatic dislocations of the hip joint are extremely rare, and only six cases have previously been reported in the literature [1, 3, 8–11]. Stein et al. [1] described anterior dislocations in younger healthy dancers. Dancers' ligaments tend to be more lax due to repetitive flexibility training, which predispose them to hip joint instability. Guyer and Levinsohn [9] reported a case of anterior dislocation due to a low-energy fall. In these cases, all the patients showed some inherent quality that predisposed them to dislocation, including anterior capsule insufficiency, small center-edge angles, and developmental dysplasia. The patient in the present case developed right hip pain and fell just after standing on her tiptoes with hip extension. We therefore diagnosed the case as that of an atraumatic dislocation.

Investigation of the probable causes of this dislocation did not reveal general joint laxity. In the fluoroscopic examination, her lower extremity was pulled manually and showed no mobility in the femoral head. Therefore, we judged from her muscle power that there was no severe muscle atrophy around her hip joint. The dislocation occurred while the patient was standing on her tiptoes with hip extension. We believed that this dislocation was not an independent event; rather, it likely occurred due to a combination of factors. Femoral neck hyperanteversion deformities can cause anterior dislocation of the hip joint. However, as the bilateral femoral neck anteversion angles in this case were −5° on CT images, a retroversion deformity of the proximal femur was revealed. The acetabular anterior coverage was measured in the sagittal plane in a standing position using a tomosynthesis imaging system. Examination revealed a VCA angle of 17° in the right hip joint in a natural standing position. The patient showed narrow acetabular coverage from the anterior area of the right femoral head. The VCA angle of right hip joint was 9.2° while standing on tiptoes with hip extension. The acetabular anterior coverage worsened with postural changes (Figure 4). Furthermore, slot radiography of the spine in the sagittal plane in a standing position revealed posterior pelvic tilt following thoracolumbar kyphosis due to vertebral fractures (Figure 3).

Based on these findings, the potential mechanism of dislocation in the present case included the following. The false-profile view revealed developmental dysplasia of the right hip joint. This dysplasia leads to increased excursion of the femoral head in the acetabulum and causes capsular laxity. This caused insufficiency of the anterior hip capsule. With development of posterior pelvic tilt following thoracolumbar kyphosis due to senile vertebral fracture, the acetabular anterior coverage worsened. When the patient with these background factors stood on her tiptoes with hip extension to pick nuts from a high place, she suffered an acute anterior dislocation of her hip joint. In the future, when vertebral fracture occurs successively and spinal kyphosis is exacerbated, there may be a risk of recurrence of the anterior dislocation of the hip joint. One cause of dislocation of the hip joint is spinal kyphosis due to vertebral fracture, as in the present case. We should not only focus on the hip joint in such cases, but also be aware of osteoporosis and observe standing sagittal spinal alignment, which are also extremely important.

The VCA angle is reportedly useful for geometric evaluation of anterior coverage in normal hips in a standing position. False-profile view radiographs are taken while the patient is standing with the pelvis rotated at 65° against the film surface. The radiograph is projected vertical to the film surface at a tube-film distance of 100 cm, with the height of the femoral head centered on the film at the level of the public symphysis. The axis of the second metatarsal bone is set parallel to the film. The VCA angle is measured from this image [12]. Chosa et al. [8] reported a cut-off value of 25° for developmental dysplasia of the hip joint. This procedure offers measurement of acetabular anterior coverage in normal hips in a standing position. However, setting posture for this procedure during radiography is complicated and the VCA angle does not indicate true anterior coverage in developmental dysplasia of hip joint [13].

Here we measured acetabular anterior coverage in the sagittal plane in a standing position using a tomosynthesis imaging system. One advantage of the tomosynthesis imaging system is reduced dose equivalents. Koyama et al. reported that the dose is 1/10 which is needed for CT [14]. Furthermore, tomosynthesis requires only a short time in a free posture. Here we photographed from the lateral side in a standing position. We believe that this procedure is not complicated compared to that of the false-profile view, and its reproducibility outperforms the other procedure. Using this method, the VCA angle of right hip joint was 17.0° in a natural standing position and 9.2° in standing on tiptoes with hip extension (Figure 4). When the patient stood on tiptoes with both hips extended to pick nuts from a high place, she likely exacerbated the developmental dysplasia of her hip joint.

In conclusion, we have described our experience with a case of atraumatic anterior dislocation of the hip joint, which was induced by activity of daily living. The possible causes of dislocation included anterior capsule insufficiency due to developmental dysplasia of the hip, posterior pelvic tilt following thoracolumbar kyphosis due to vertebral fracture, and acetabular anterior coverage changes based on postural changes. The changes in acetabular anterior coverage due to postural changes were measured in the sagittal plane using a tomosynthesis imaging system. The tomosynthesis imaging system was useful for elucidation of mechanism of dislocation in the present case.
